# First description of the male of *Troglocoelotes doul* Lin & Li, 2024 (Araneae, Agelenidae, Coelotinae)

**DOI:** 10.3897/BDJ.14.e214835

**Published:** 2026-10-02

**Authors:** Junshuo Wang, Mian Wei, He Zhang, Jie Liu, Changhao Hu

**Affiliations:** 1 Arachnid Resource Centre of Hubei Province & Hubei Key Laboratory of Regional Development and Environmental Response, Faculty of Resources and Environmental Science, Hubei University, Wuhan, China Arachnid Resource Centre of Hubei Province & Hubei Key Laboratory of Regional Development and Environmental Response, Faculty of Resources and Environmental Science, Hubei University Wuhan China; 2 College of Life Sciences, Sichuan University, Chengdu, China College of Life Sciences, Sichuan University Chengdu China; 3 College of Physics and Electronic Engineering, Xingtai University, Xingtai, China College of Physics and Electronic Engineering, Xingtai University Xingtai China; 4 Centre for Behavioral Ecology and Evolution, School of Life Sciences, Hubei University, Wuhan, China Centre for Behavioral Ecology and Evolution, School of Life Sciences, Hubei University Wuhan China; 5 College of Life Sciences, Hunan Normal University, Changsha, China College of Life Sciences, Hunan Normal University Changsha China

**Keywords:** cave, morphology, spider, taxonomy, troglobite

## Abstract

**Background:**

*Troglocoelotes* Zhao & Li, 2019 is currently known from Guangxi and Guizhou, China and comprises 12 species. Of these, only four are known from both sexes, whereas the remaining eight are known only from females. *Troglocoelotes doul* Lin & Li, 2024 was originally described from a female specimen collected in Guangxi and its male has remained unknown.

**New information:**

Based on specimens collected from the type locality of *Troglocoelotes doul* Lin & Li, 2024, the male of this species is described for the first time.

## Introduction

*Troglocoelotes* Zhao & Li, 2019 is currently the only genus of Coelotinae F. O. Pickard-Cambridge, 1893 composed entirely of troglobitic species (Zhou et al. 2023). Members of *Troglocoelotes* exhibit typical cave-associated morphological characteristics, including reduction or loss of pigmentation and regression or loss of eyes (Mammola and Isaia 2017, Li et al. 2019). They inhabit the deep zones of limestone caves in Guangxi and Guizhou, China, where they build webs in rock crevices, shallow depressions on the cave floor and beneath loose stones (personal observation). To date, 12 extant species have been described in the genus, eight of which are known only from females (World Spider Catalog 2026).

Shenlonggong Cave is located in Huanjiang, Guangxi, an area characterised by extensive karst landscapes and numerous limestone caves. The cave is the type locality of several troglobitic species, including *Coecobrya draconis* Zhang & Dong, 2014 (Collembola Lubbock, 1871), *Desmoxytes scolopendroides* Golovatch, Geoffroy & Mauriès, 2010 (Diplopoda Blainville in Gervais, 1844), *T. doul* Lin & Li, 2024 (Araneae Clerck, 1757), amongst others (Golovatch et al. 2010, Zhang and Dong 2014, Lin et al. 2024).

*Troglocoelotes doul* was originally described, based on a female specimen and its male has remained unknown (Lin et al. 2024). During the examination of recently collected spider specimens from the cave, one female was identified as *T. doul* and two males were considered conspecific, based on their co-occurrence and morphological similarities, particularly their similarly degenerated eyes. The male of *T. doul* is, therefore, described herein for the first time.

## Materials and methods

The specimens examined in this study are deposited in the Centre for Behavioral Ecology and Evolution (CBEE), School of Life Sciences, Hubei University in Wuhan, China (curator: Jie Liu). Specimens were examined using an OLYMPUS SZX7 stereomicroscope. The male palps were examined and photographed after dissection. The epigyne was dissected from the body, immersed in 0.1 mg/ml Protease K solution, heated in a water bath at 56℃ and stained with Amido black 10B. All measurements were taken using a Leica M205 C stereomicroscope. Eye diameters were taken at the widest point. Leg measurements are given as the total length of leg (femur, patella, tibia, metatarsus, tarsus). All measurements are in millimetres (mm). Photographs were taken using Leica M205 C stereomicroscope and OLYMPUS BX51 microscope and multifocal images were produced using Helicon Focus (Version 8.1.0).

Abbreviations: ALE–anterior lateral eye; AME–anterior median eye; PLE–posterior lateral eye; PME–posterior median eye; I, II, III, IV–legs I to IV.

## Taxon treatments

### Troglocoelotes doul

Lin & Li, 2024

5ADAF947-46FA-53B8-8790-98F8856B7E12

Troglocoelotes doul Lin & Li, in Lin et al. (2024): 7, figs. 1A, B, 3A and 4.

#### Materials

- **Type status:** Holotype. **Occurrence:** recordedBy: Haolin Mo and Shanmi Zheng; individualCount: 1; sex: female; lifeStage: adult; **Location:** country: China; stateProvince: Guangxi Zhuang Autonomous Region; county: Hechi City, Huanjiang Maonan Autonomous County; locality: Dacai Township, Shenlonggong Cave; verbatimLatitude: 108.3898°E; verbatimLongitude: 24.7707°N; **Identification:** identificationRemarks: not examined; **Event:** year: 2023; month: 8; day: 1; **Record Level:** institutionCode: IZCAS-Ar44790
- **Type status:** Other material. **Occurrence:** recordedBy: Changhao Hu, Jian Chang, Jinhui Wang, and Mingwei Li; individualCount: 3; sex: 2 males, 1 female; lifeStage: adult; **Location:** country: China; stateProvince: Guangxi Zhuang Autonomous Region; county: Hechi City, Huanjiang Maonan Autonomous County; locality: Dacai Township, Shenlonggong Cave; verbatimElevation: 368 m; verbatimLatitude: 24.7707°N; verbatimLongitude: 108.3898°E; **Event:** year: 2026; month: 8; day: 10

#### Description

**Figure 1.**
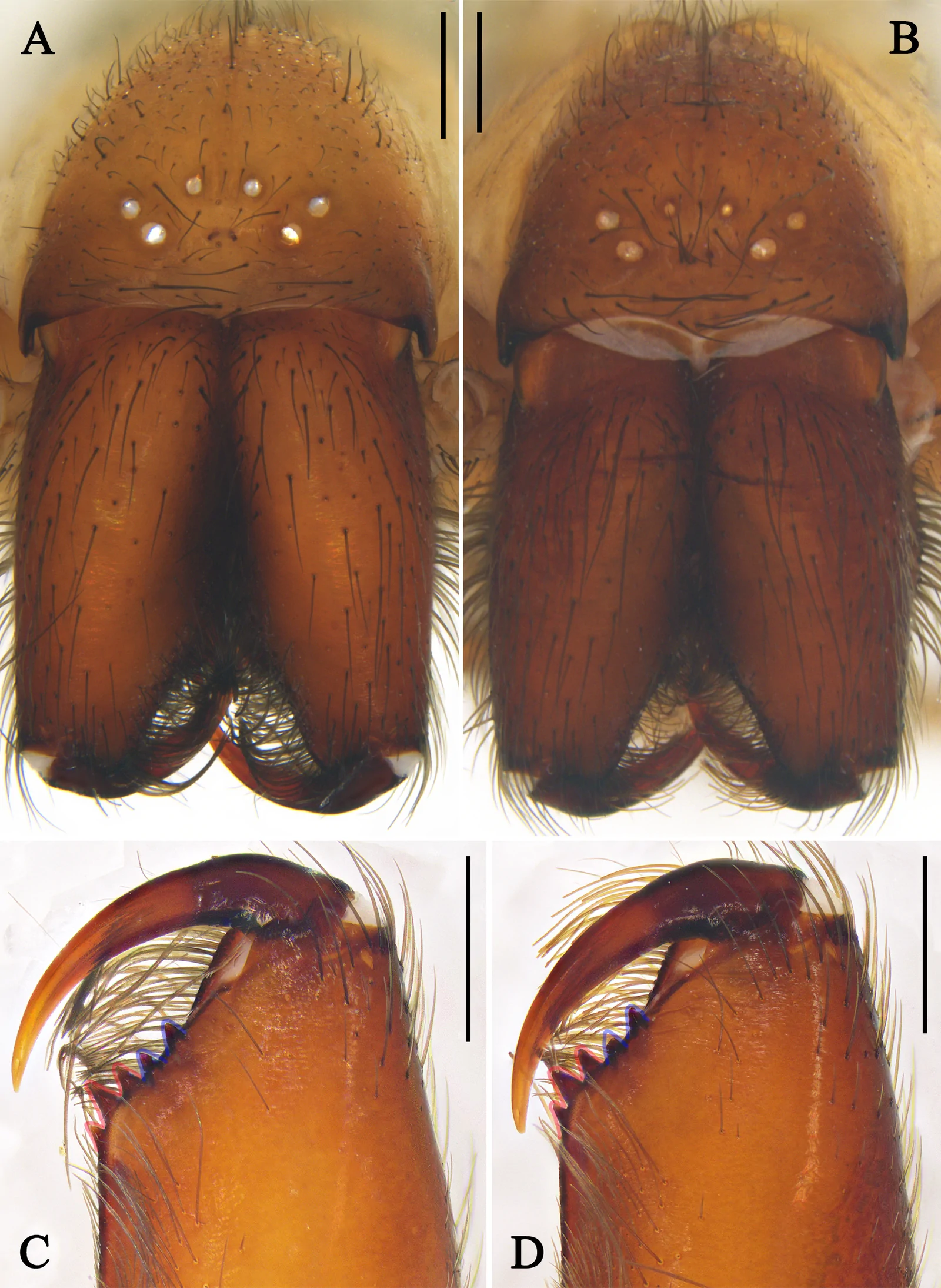
*Troglocoelotes doul* Lin & Li, 2024. **A, B** eye areas, frontal view; **C, D** chelicerae, retrolateral view (red lines: promarginal teeth; blue lines: retromarginal teeth); **A, C** male; **B, D** female. Scale bars: 0.5 mm.

**Figure 2.**
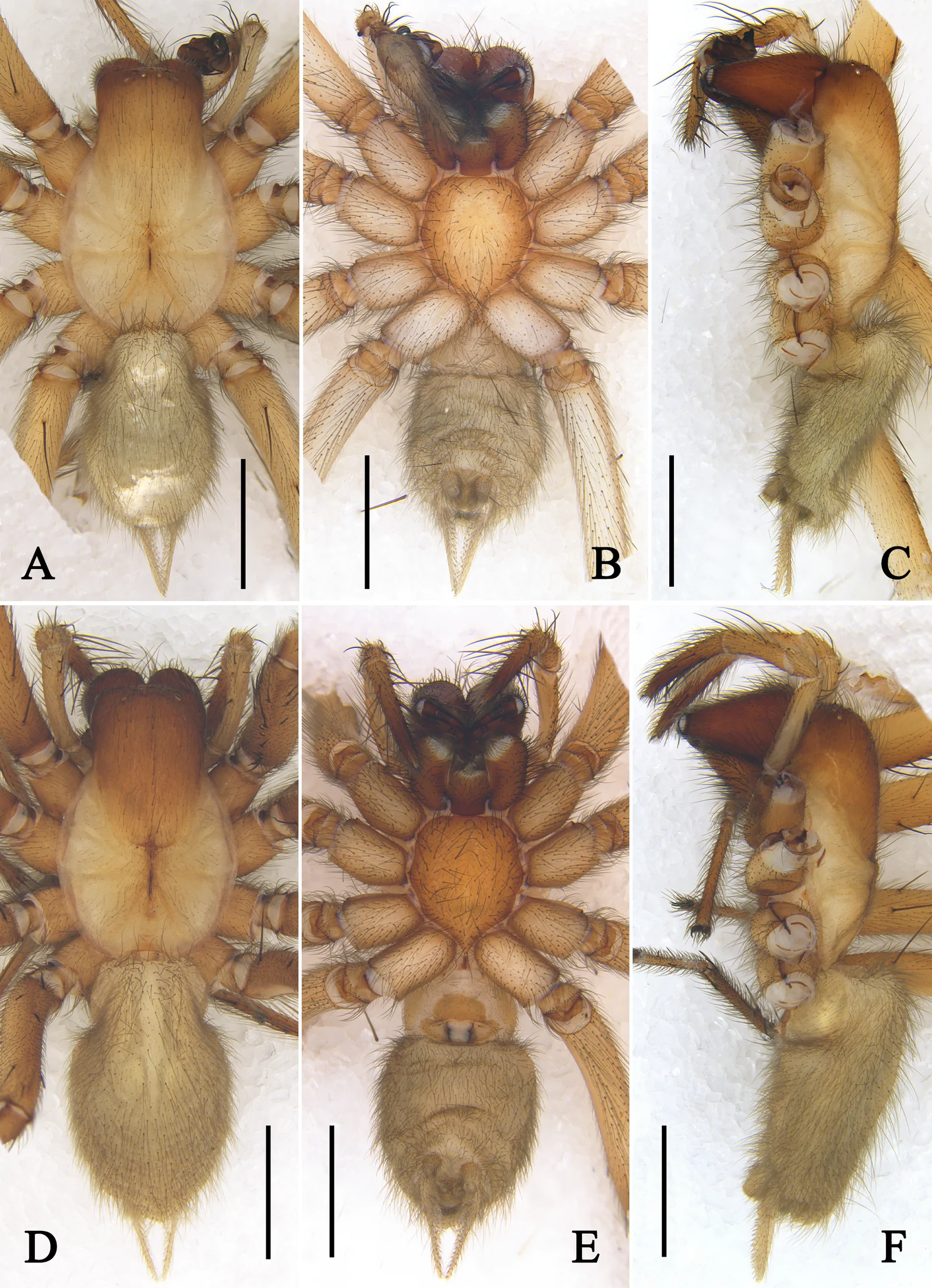
Habitus of *Troglocoelotes doul* Lin & Li, 2024. **A–C** male; **D–F** female; **A, D** dorsal view; **B, E** ventral view; **C, F** lateral view. Scale bars: 2 mm.

**Male**: Total length 7.19, carapace 4.19 long, 2.74 wide, opisthosoma 3.12 long, 2.05 wide. Eyes vestigial (Fig. 1A). Eye sizes and interdistances: AME absent, ALE 0.07, PME 0.06, PLE 0.09, PME–PME 0.16, PME–PLE 0.19, ALE–PME 0.16, ALE–PLE 0.07. Leg measurements: I 17.25 (4.30, 1.06, 4.53, 4.37, 2.99), II 15.98 (4.23, 1.03, 3.94, 4.10, 2.68), III 15.30 (3.96, 0.92, 3.58, 4.24, 2.60), IV 19.94 (4.85, 1.12, 4.80, 6.15, 3.02). Leg formula IV-I-II-III. Chelicerae with three promarginal and two retromarginal teeth (Fig. 1C). Opisthosoma and legs with long hairs (Fig. 2A–C).

Colouration (Fig. 2A–C). Carapace yellowish-brown, head region light brown, thoracic region pale yellow, fovea black. Chelicerae dark brown. Endites and labium brown, with white distal parts. Sternum yellowish-brown. Legs light brown. Opisthosoma and spinnerets grey.

**Figure 3.**
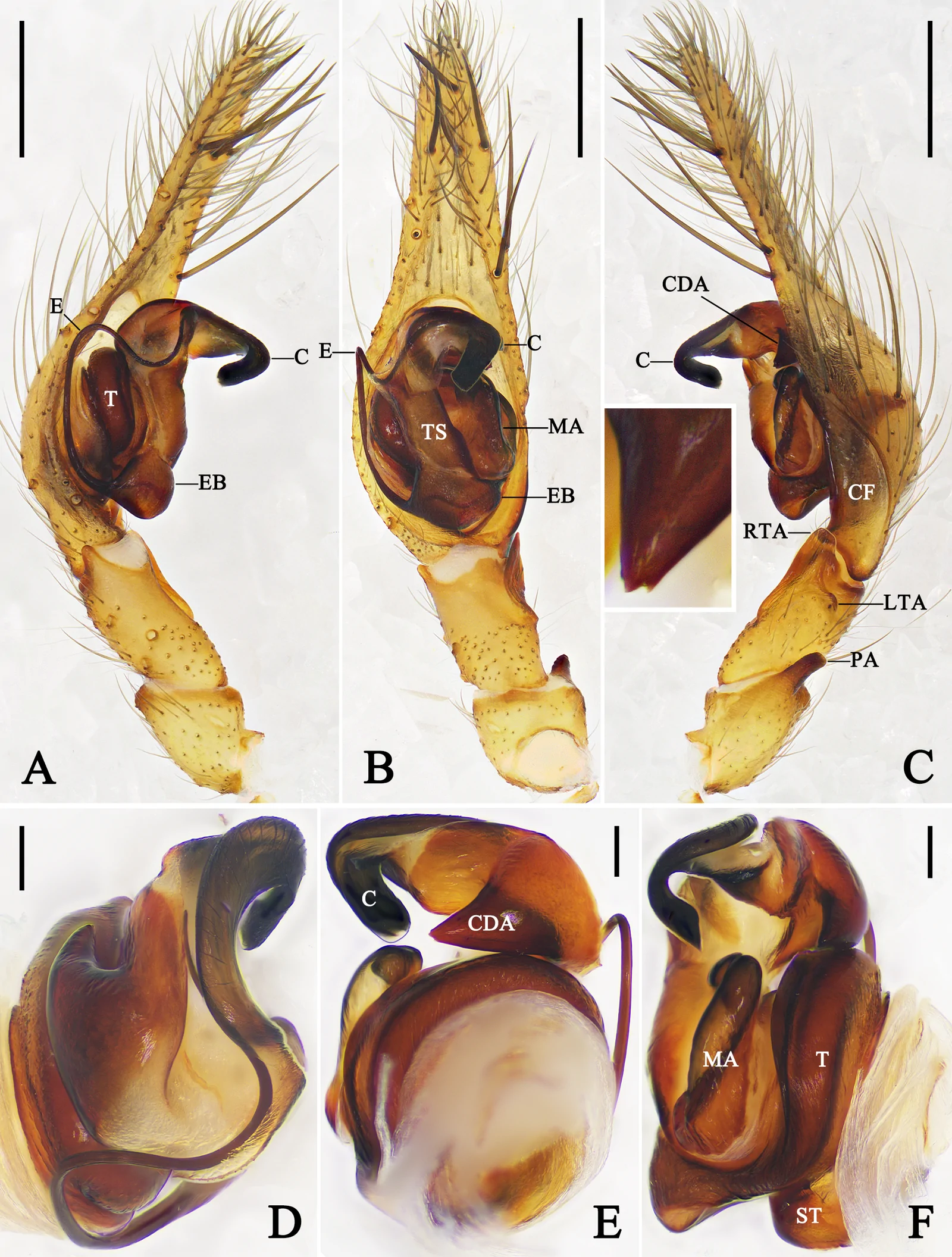
Male palp of *Troglocoelotes doul* Lin & Li, 2024. **A–C** left palp (**A** prolateral view; **B** ventral view; **C** retrolateral view, inset showing the denticles on the distal tip of CDA); **D–F** right bulb, horizontally flipped (**D** apical view; **E** dorsal view; **F** retrolateral view). Abbreviations: C: conductor; CDA: dorsal apophysis of conductor; CF: cymbial furrow; E: embolus; EB: embolic base; LTA: lateral tibial apophysis; MA: median apophysis; PA: patellar apophysis; RTA: retrolateral tibial apophysis; ST: subtegulum; T: tegulum; TS: tegular sclerite. Scale bars: 0.5 mm (A–C), 0.1 mm (D–F).

Palp (Fig. 3). Patellar apophysis digitiform. Retrolateral tibial apophysis trapeziform, almost 2/3 the length of tibia, with its distal end slightly extending beyond the tibia. Lateral tibial apophysis short and rounded. Cymbium slender, more than twice as long as bulb; cymbial furrow almost 1/3 the length of cymbium. Median apophysis spoon-shaped, almost half the length of bulb. Conductor strong and sclerotised, curved ventrally, with weak basal lamellar and blunt end; dorsal apophysis of conductor triangular, distal tip with three denticles. Embolus filform and curved, arising from embolic base at 8:30 o’clock position.

**Figure 4.**
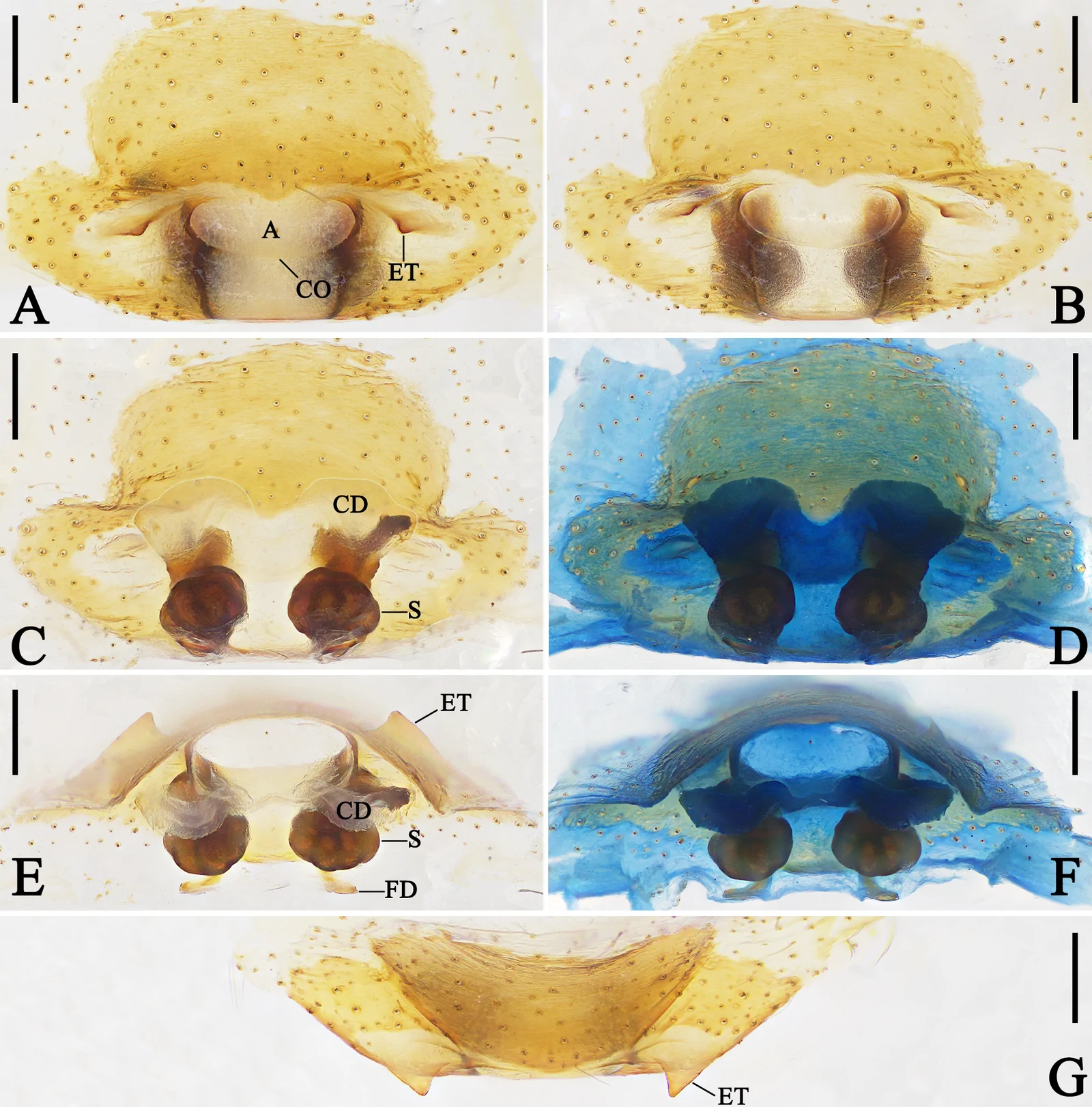
Female genitalia of *Troglocoelotes doul* Lin & Li, 2024. **A, B** epigyne, ventral view (**A** uncleaned; **B** cleaned); **C, D** vulva, dorsal view (**C** unstained; **D** stained); **E, F** vulva, anterodorsal view (**E** unstained; **F** stained); **G** epigyne, anteroventral view. Abbreviations: A: atrium; CD: copulatory duct; CO: copulatory opening; ET: epigynal tooth; FD: fertilisation duct; S: spermatheca. Scale bars: 0.2 mm.

**Female**: See Lin et al. (2024) (Fig. 1B, D, Fig. 2D–F and Fig. 4).

#### Diagnosis

**Figure 5.**
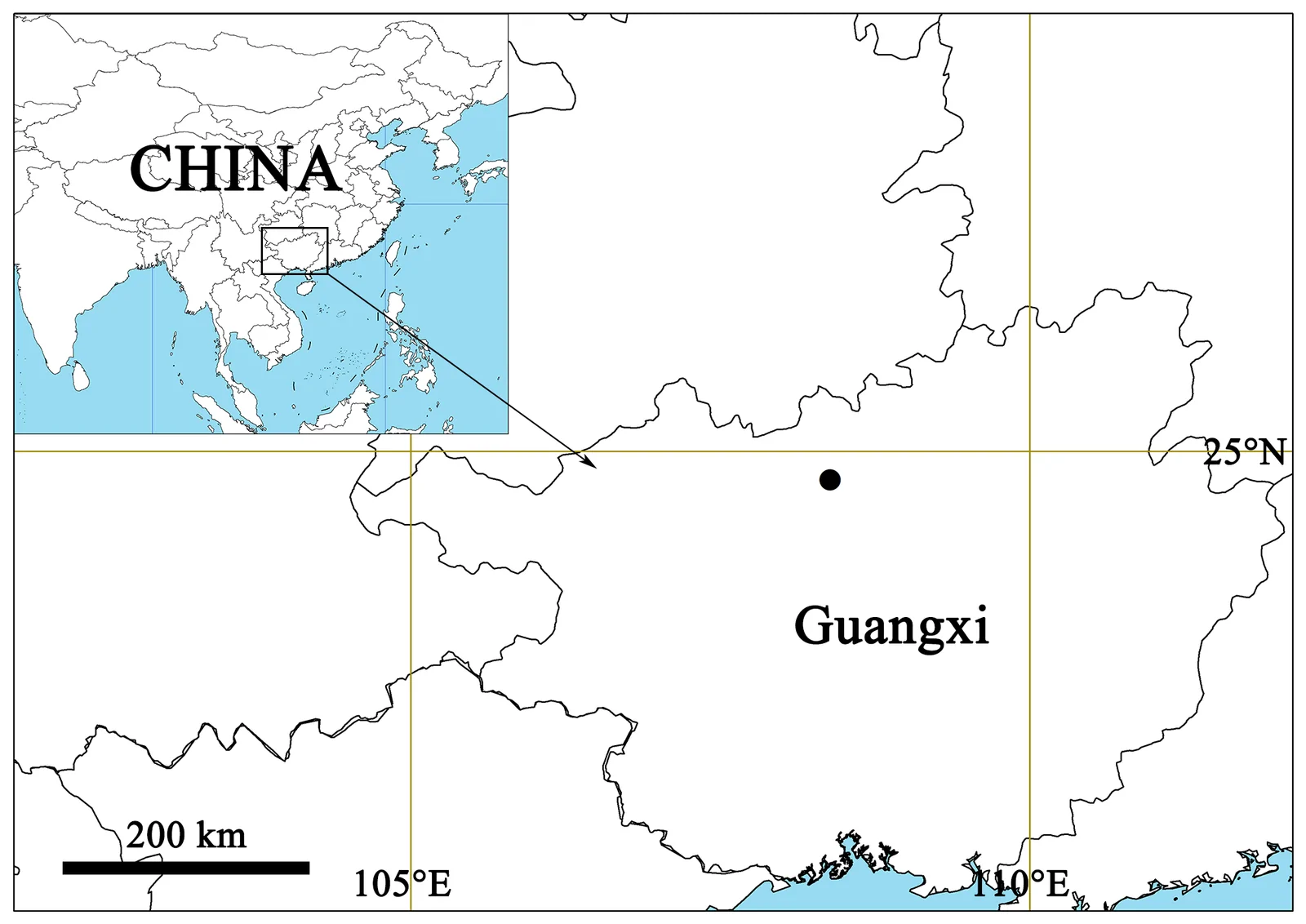
Distribution map of *Troglocoelotes doul* Lin & Li, 2024.

The male of *T. doul* can be distinguished from those of all other male-known congeners by the following combination of characters: 1. cymbium more than twice as long as bulb (less than twice as long as bulb in all other male-known congeners); 2. patella apophysis digitiform (pointed in *T. tortus*); 3. median apophysis large, almost half the length of bulb (almost 1/3 the length of bulb in all other male-known congeners); 4. conductor curved, with blunt distal end (straight and pointed in all other male-known congeners); 5. dorsal apophysis of conductor triangular and pointed (rounded and blunt in *T. qixianensis* and *T. tortus*, absent in *T. liangensis* and *T. yumiganensis*); 6. embolus arising from embolic base at 8:30 o’clock position (6 o’clock position in all other male-known congeners) (cf. Fig. 3 and figs. 5, 8, 10 and 12 in Li et al. (2019)).

The female of this species is similar to those of *T. sinanensis* Zhou & Zhao, 2023 in having wide copulatory opening, the posterior margin of atrium almost as wide as atrium and the epigynal teeth situated laterally on atrium (cf. Fig. 4 and figs. 3A–C in Zhou et al. (2023)), but can be distinguished by: 1. lateral part of copulatory duct with a projection (absent in *T. sinanensis*); and 2. spermatheca spherical, narrower than copulatory duct (oval and laterally elongated, wider than copulatory duct in *T. sinanensis*).

#### Distribution

Known only from type locality (Shenlonggong Cave in Guangxi, China) (Fig. 5).

## Supplementary Material

XML Treatment for Troglocoelotes doul
